# Bias Introduced by Multiple Head Coils in MRI Research: An 8 Channel and 32 Channel Coil Comparison

**DOI:** 10.3389/fnins.2019.00729

**Published:** 2019-07-15

**Authors:** Jessica L. Panman, Yang Yang To, Emma L. van der Ende, Jackie M. Poos, Lize C. Jiskoot, Lieke H. H. Meeter, Elise G. P. Dopper, Mark J. R. J. Bouts, Matthias J. P. van Osch, Serge A. R. B. Rombouts, John C. van Swieten, Jeroen van der Grond, Janne M. Papma, Anne Hafkemeijer

**Affiliations:** ^1^Department of Radiology, Leiden University Medical Center, Leiden, Netherlands; ^2^Department of Neurology, Erasmus University Medical Center Rotterdam, Rotterdam, Netherlands; ^3^Department of Methodology and Statistics, Institute of Psychology, Leiden University, Leiden, Netherlands; ^4^Leiden Institute for Brain and Cognition, Leiden University, Leiden, Netherlands

**Keywords:** MRI, DTI, neuroimaging, gray matter, white matter, multicenter study, bias

## Abstract

Neuroimaging MRI data in scientific research is increasingly pooled, but the reliability of such studies may be hampered by the use of different hardware elements. This might introduce bias, for example when cross-sectional studies pool data acquired with different head coils, or when longitudinal clinical studies change head coils halfway. In the present study, we aimed to estimate this possible bias introduced by using different head coils to create awareness and to avoid misinterpretation of results. We acquired, with both an 8 channel and 32 channel head coil, T1-weighted, diffusion tensor imaging and resting state fMRI images at 3T MRI (Philips Achieva) with stable acquisition parameters in a large group of cognitively healthy participants (*n* = 77). Standard analysis methods, i.e., voxel-based morphometry, tract-based spatial statistics and resting state functional network analyses, were used in a within-subject design to compare 8 and 32 channel head coil data. Signal-to-noise ratios (SNR) for both head coils showed similar ranges, although the 32 channel SNR profile was more homogeneous. Our data demonstrates specific patterns of gray and white matter volume differences between head coils (relative volume change of 6 to 9%), related to altered image contrast and therefore, altered tissue segmentation. White matter connectivity (fractional anisotropy and diffusivity measures) showed hemispherical dependent differences between head coils (relative connectivity change of 4 to 6%), and functional connectivity in resting state networks was higher using the 32 channel head coil in posterior cortical areas (relative change up to 27.5%). This study shows that, even when acquisition protocols are harmonized, the results of standardized analysis models can be severely affected by the use of different head coils. Researchers should be aware of this when combining multiple neuroimaging MRI datasets, to prevent coil-related bias and avoid misinterpretation of their findings.

## Introduction

Large multicenter data samples are increasingly used to establish and reproduce MRI neuroimaging findings. Although pooling MR imaging data contributes to increased study power, the reliability and results from such studies may be compromised by the use of different hardware elements (Cannon et al., 2014; Smith and Nichols, 2018). For example, changing head coils during a longitudinal study, or combining cross-sectional data acquired with different head coils may introduce a coil-related bias (Focke et al., 2011; Pardoe et al., 2016). Studies that depict the effect of using multiple head coils are currently limited to analysis of T1 weighted imaging data (Focke et al., 2011; Pardoe et al., 2016), demonstrating a difference in gray matter volume (Focke et al., 2011), and cortical thickness (Pardoe et al., 2016). Still, the effects on other quantitative MRI variables, for instance obtained with diffusion tensor imaging or resting state functional MRI, are unknown, but highly important for multicenter or longitudinal studies using different types of hardware. Identification of the brain regions affected by coil-related bias is essential, not only to increase awareness, but more importantly to avoid misinterpretation of results from studies using multiple MRI hardware elements (Li et al., 2018). In the present study, we aimed to estimate and depict the impact of using different receive-only phased array head coils (8 channel head coil and 32 channel head coil) on T1 weighted, DTI and resting state functional MRI data using a within-subject design in a large cohort of cognitively healthy subjects.

## Materials and Methods

### Study Procedure and Participants

For the present study, we included 77 cognitively healthy participants who underwent MRI of the brain on a 3Tesla Philips Achieva scanner (Philips Medical Systems, Best, Netherlands) at the Leiden University Medical Center, Leiden, Netherlands. The MRI protocol contained T1 weighted, DTI and resting state functional MR images, acquired with both an 8 channel SENSE head coil (8CH) and an 32 channel SENSE head coil (32CH) within one MRI session (coil geometry is displayed in Supplementary Figure S1). During acquisition, optimal image quality was obtained by using the incorporated “constant level appearance” (CLEAR) inhomogeneity correction algorithm on the scanner. We display one raw dataset for all sequences from both coils from a representative healthy participant in Supplementary Figure S2.

Cognitively healthy participants were included in the context of the prospective longitudinal frontotemporal dementia risk cohort (FTD-RisC) in which families with autosomal dominant inherited FTD gene mutations are followed using standardized assessment protocols including an MRI of the brain every year, as described previously (Dopper et al., 2014; Papma et al., 2017). To confirm cognitively healthy status of all participants, Mini Mental State Examination [MMSE (Folstein et al., 1975)] and the Frontal Assessment Battery [FAB (Dubois et al., 2000)] are reported as cognitive screening measures and the Neuropsychiatric Inventory [NPI-Q (Cummings et al., 1994)] and Frontotemporal Dementia Rating Scale [FRS (Mioshi et al., 2010)] are reported as behavioral screening questionnaires.

### MRI Acquisition

#### Signal to Noise Assessment

For signal to noise ratio’s (SNR) assessment, we acquired proton density weighted single-slice images with one noise-only image, i.e., without radiofrequency pulses, in one healthy volunteer, following the procedures described by Wiggins et al. (2006). For both coils, we acquired the images in the axial, coronal and sagittal direction, with the following parameters: Repetition time (TR) = 200 ms, echo time (TE) = 3.1 ms, field of view (FOV) = 220 × 220 × 3 mm, flip angle = 20° (for noise scan 0°), slice thickness = 3 mm, voxel size 0.85 × 0.85 × 3.0 mm, number of averages = 10, acquisition time = 9 min and 24 s.

#### T1 Weighted Imaging

For the 3DT1 weighted acquisition, scanning parameters were as follows: MPRAGE, TR = 9.7 ms, TE = 4.6 ms, FOV = 224 × 177 × 168 mm, flip angle = 8°, slices = 140, voxel size = 0.88 × 0.88 × 1.2 mm, SENSE = none, acquisition time = 4 min and 56 s. Identical parameters were used for both the 8CH and the 32CH coils.

#### Diffusion Imaging

Diffusion imaging was performed in 60 non-collinear gradient directions using single shot echo planar imaging. The phase encoding direction was anterior to posterior for both coils. The following parameters were used for the 8CH coil: 60 *b* = 1000 s/mm^2^, TR = 8250 ms, TE = 80 ms, FOV = 256 × 208 × 140 mm, flip angle = 90°, slices = 70, voxel size = 2 × 2 × 2 mm, SENSE = 2.0, one *b* = 0 s/mm2 acquisition, scan time = 8 min and 48 s, 2 signal averages. For the 32CH coil, we increased the number of slices to 80, to have sufficient coverage to include the cerebellum in the imaging volume for our longitudinal FTD-RisC study (Papma et al., 2017). As a result, the TR for the 32CH coil was 9245 ms, FOV was 256 × 232 × 160 mm, and acquisition time increased to 9 min and 52 s with a SENSE factor of 2.3. Other parameters were identical between both head coils.

#### Resting State Functional MRI

For resting state fMRI, T2^*^-weighted images were acquired using whole brain multislice gradient echo planar imaging. For both coils, the following parameters were used: TR = 2200 ms, TE = 30 ms, FOV = 220 × 220 × 113 mm, flip angle = 80°, slices = 38, voxel size = 2.75 × 2.75 × 2.99 mm, including 10% interslice gap, SENSE = 3.0, volumes = 200, acquisition time = 7 min and 28 s. Participants were instructed to lie still with their eyes closed and stay awake during the resting state fMRI scans.

### MRI Processing

Before image preprocessing and analysis, we checked the scans thoroughly for image quality and the presence of artifacts. Data processing and statistical analyses were carried out using Functional Magnetic Resonance Imaging of the Brain Software Library (FSL) version 5.0.8. (Jenkinson et al., 2012).

#### Signal to Noise Ratio

For both the 8CH coil and 32CH coil, we isolated an average signal image and one noise-only image for each orientation plane. We subdivided the signal and noise images into non-overlapping regions of interest (ROI) of 16 by 16 voxels. Next, we calculated the mean signal of the ROI using the averaged signal image, and the standard deviation of the noise of the ROI using the noise-only image. Since the noise images were amplitude-reconstructed, the measured standard deviation was corrected for the Rician noise distribution (Haacke et al., 1999). Ultimately, for each ROI, SNR was calculated according to:

$$\mathrm{SNR}=\frac{\mathrm{Mean}\,\mathrm{Signal}}{\sqrt{\frac{2}{4-\pi }}\times \mathrm{Std}.\mathrm{Noise}}$$

Third, we translated the SNR ROI matrices into color-coded maps, in order to visualize the SNR distribution throughout the brain for both coils.

#### Structural Imaging

To assess the influence of the head coil on gray and white matter volume measurements, we applied the standard voxel-based morphometry (VBM) pipeline as implemented in FSL. Preprocessing of the T1 weighted images included brain extraction followed by radiofrequency (RF) inhomogeneity correction, tissue segmentation and realignment to Montreal Neurological Institute (MNI) standard space using non-linear registration. We performed quality control to ensure good brain extraction, that was not different between both head coils. Next, FMRIB’s Automated Segmentation Tool (FAST) was used for correction for spatial intensity variations, also known as bias field or RF inhomogeneity, and segmentation of the T1 weighted images (Zhang et al., 2001). The corrected, segmented gray matter images were re-registered non-linearly to a study-specific template with a balanced set of 8CH and 32CH coil images. The registered partial volume images were divided by the Jacobian of the warp field to correct for any local expansion or contraction. An isotropic Gaussian kernel with a sigma of 3 mm, which corresponds to a full width at half maximum kernel (FWHM) of approximately 7 mm, was used to smooth the gray matter segmentations. We also applied the VBM processing pipeline to the white matter segmentations, resulting in registered, corrected and smoothed white matter images for voxel-wise analyses.

#### Diffusion Imaging

Diffusion scans were corrected for motion artifacts and eddy currents by alignment to the *b* = 0 image using the FMRBIB Diffusion Toolbox. The tensor was fitted each voxel to create fractional anisotropy (FA) and mean diffusivity (MD) images. Subsequently, we applied standard tract-based spatial statistics (TBSS) as implemented in FSL (Smith et al., 2006). FA images were aligned to standard space using non-linear registration and averaged into a mean FA image. To create a study-specific FA mask, we thresholded the mean FA image with a minimum value of FA ≥ 0.2. This binarised FA mask was applied to voxel-wise comparisons of FA and MD between coils.

#### Resting State Functional MRI

For preprocessing of the resting state fMRI scans, we applied the fMRI Expert Analysis Tool (FEAT) as implemented in FSL, consisting of motion correction with MCFLIRT and spatial smoothing with a kernel of 6 mm FWHM. The data-driven independent component analysis (ICA) based Automatic Removal of Motion Artifacts (ICA-AROMA) approach was used to identify and remove noise components from the resting state fMRI data (Pruim et al., 2015). After denoising, high pass temporal filtering was performed with a cut-off frequency of 0.01 Hz. The functional resting state images were registered to the corresponding T1 weighted images using Boundary-Based Registration and were subsequently registered to the 2 mm isotropic MNI standard space using non-linear registration with a warp resolution of 10 mm. Voxel-based functional connectivity was studied in a standardized manner using the eight standard Beckmann resting-state functional networks of interest (Beckmann et al., 2005), i.e., the medial and lateral visual system network, the primary auditory network – also known as the salience network –, the sensory motor network, the default mode network, the executive control network and the left and right dorsal visual processing stream networks. To further account for noise, white matter and CSF templates were included in the analyses as regressors. Functional connectivity of each network of interest was calculated using dual regression, as previously described (Hafkemeijer et al., 2017). In short, the eight standard resting state networks (Beckmann et al., 2005) were used as a reference. Voxel-based resting state functional connectivity was determined in terms of similarity of the BOLD fluctuations in the brain in relation to characteristic fluctuations in the standard resting state networks. With dual regression, individual time series were first extracted for each template, using the resting state networks, and the two additional white matter and cerebrospinal fluid maps, in a spatial regression against the individual fMRI data set (regression 1). The resulting matrices described temporal dynamics for each template and individual. Next, the temporal regressors were used to fit a linear model to the individual fMRI data set (regression 2), to estimate the spatial maps for each individual. This results in 3D images for each individual, with voxel-wise *z*-scores representing the functional connectivity to each of the predefined standard networks.

#### Statistical Analysis

For all analyses, we designed within-subject paired sample *t*-tests with each subject’s mean effect to analyze head coil differences in gray and white matter volume, FA, MD, and resting state functional connectivity. We performed voxel-based non-parametric permutation testing (Nichols and Holmes, 2002) with 5000 permutations using FSL-randomize. The statistical threshold was set at *p* < 0.05, using threshold-free cluster enhancement (TFCE) technique and family-wise error (FWE) correction to correct for multiple comparisons across voxels. We quantified the severity of head coil differences by calculating effect sizes and percentage of change.

#### Voxel-Specific Scaling Factors

For our own longitudinal clinical study (Dopper et al., 2014; Papma et al., 2017), we aimed to create voxel-based scaling factors to correct for the use of different head coils. For the T1-weighted, DTI and resting state fMRI images, we calculated and validated voxel-specific scaling images. Procedures are described in detail in the Supplementary Material. In short, we separated our sample into a template dataset (*n* = 39) and validation dataset (*n* = 38), matched for age and sex. For the template dataset, we merged and averaged the images into a mean 8CH coil image and 32CH coil image. The averaged 32CH coil image was divided by the averaged 8CH coil image, resulting in a voxel-based scaling factor. We reduced noise by applying a median filter with a kernel of 5 mm. Next, the 8CH coil images from the validation set were multiplied with the voxel-based scaling factor, equalizing the 8CH coil images to the signal intensity of the 32CH coil images. We repeated previous described statistical analysis on the validation set to complete verification of the scaling factor.

## Results

### Participants

In total 77 participants were included in this study (Table 1). Cognitive and behavioral screening tests confirmed a cognitively healthy status of all participants.

**TABLE 1:** Sample characterization.

Age, years	54.2 (28–76)
Sex, female/male	54/23
Education^a^	5.29 (1–7)
MMSE^b^	29.3 (25–30)
FAB^b^	16.8 (11–18)
NPI^b^	1.6 (0–18)
FRS^b^	96.4 (73–100)

*Values are means (range) for continuous variables and ratio for dichotomous variables. Abbreviations: MMSE = Mini-Mental State Examination; FAB = Frontal Assessment Battery; NPI = Neuropsychiatric Inventory; FRS = Frontotemporal Dementia Rating Scale. ^*a*^Education is presented on a 7-point scale ranging from 1 (less than elementary school) to 7 (university or technical college) according to Verhage (1964). ^*b*^Missing data: MMSE 1/77, FAB 1/77, NPI 17/77, FRS 16/77.*

### Signal to Noise Ratio

**FIGURE 1:**
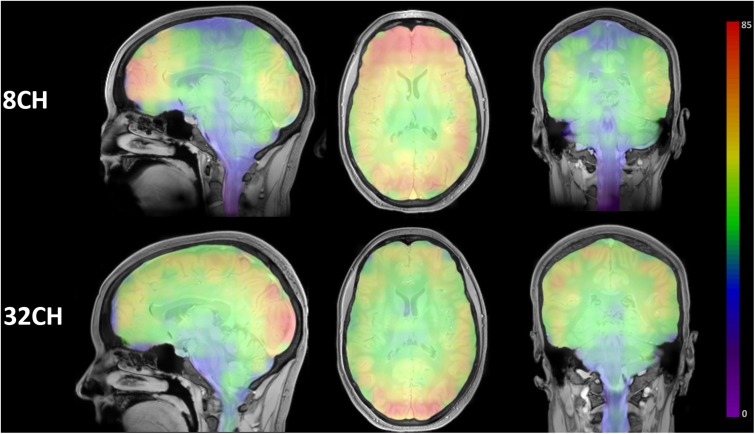
Color-coded SNR brain maps in the sagittal, axial and coronal direction for 8 channel head coil (8CH) and 32 channel head coil (32CH). Colorbar represents SNR values, ranging 0-85.

Visualization of the SNR ROI matrices revealed a quite homogeneous distribution of SNR throughout the brain using the 32CH coil, with the highest SNR in posterior cortical areas of the brain. SNR of the 8CH coil was highest in the frontal lobe, but dropped in central and medial areas (Figure 1).

### Structural Imaging

**FIGURE 2:**
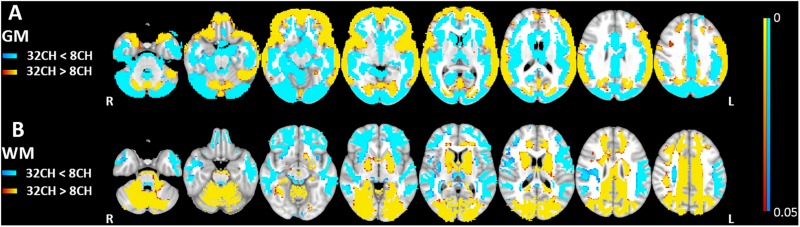
Voxel-based coil differences in panel **(A)** gray matter (GM) and **(B)** white matter volume (WM) using voxel-based morphometry paired sample *t*-tests on T1 weighted images. 8CH = 8 channel head coil, 32CH = 32 channel head coil. *p* values are color coded from 0.05 to <0.0001 FWE corrected.

Quality control showed no differences in brain extraction of the T1 weighted images between the 8CH and 32CH coil. Gray matter volumes obtained with the 32CH coil were larger than obtained with the 8CH coil (*p*^FWE^ < 0.05, effect size = 2.096, increase = 6.2%), particularly in the middle and inferior frontal lobe, the superior and middle temporal lobe, the anterior insular cortex, the temporo-parietal junction, the paracingulate, and the cuneus (yellow areas in Figure 2A). Gray matter volumes appeared smaller using the 32CH coil (*p*^FWE^ < 0.05, effect size = 2.571, decrease = 8.9%) in frontal and deeper cerebral areas, such as the medial temporal lobe, medial frontal lobe, basal ganglia, posterior insular cortex, anterior cingulate, superior frontal cortex, occipital lobe, and the cerebellum (blue areas in Figure 2A). In the white matter, we found larger white matter volumes in subcortical and posterior cortical regions using the 32CH coil compared with the 8CH coil (*p*^FWE^ < 0.05, effect size = 1.951, increase = 8.5%; see yellow areas in Figure 2B). White matter volumes were smaller using the 32CH coil in frontotemporal regions (*p*^FWE^ < 0.05, effect size = 1.637, decrease = 6.1%; see blue areas in Figure 2B).

### Diffusion Tensor Imaging

**FIGURE 3:**
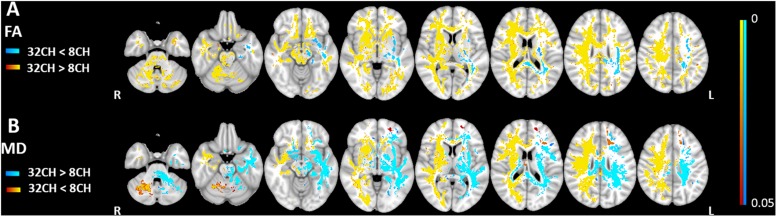
Voxel-based coil differences in panel **(A)** fractional anisotropy (FA) and **(B)** mean diffusivity (MD) using tract-based spatial statistics paired sample *t*-test on diffusion weighted images. 8CH = 8 channel head coil, 32CH = 32 channel head coil. *p* values are color coded from 0.05 to <0.0001 FWE corrected.

We found higher FA values (*p*^FWE^ < 0.05, effect size = 2.197, increase = 5.7%) with the 32CH coil compared with the 8CH coil in all tracts of the right hemisphere and frontal tracts of the left hemisphere, such as the forceps minor, anterior parts of the uncinate fasciculus, anterior thalamic radiation, inferior fronto-occipital fasciculus, and superior longitudinal fasciculus (yellow areas in Figure 3A). On the contrary, we found lower FA values using the 32CH coil (*p*^FWE^ < 0.05, effect size = 2.038, decrease = 5.0%) in part of the left-sided posterior tracts, such as the forceps major, the corticospinal tract, the inferior longitudinal fasciculus, and the central and posterior parts of the anterior thalamic radiation (blue areas in Figure 3A). MD was lower using the 32CH coil compared to 8CH coil (*p*^FWE^ < 0.05, effect size = 1.871, decrease = 4.6%) in the entire right hemisphere, and some tracts of the left hemisphere located in the prefrontal and the occipital lobe (red-yellow areas in Figure 3B). Using the 32CH coil, MD was higher (*p*^FWE^ < 0.05, effect size = 1.952, increase = 4.7%) in all tracts of the left hemisphere, except for the prefrontal and occipital lobe (blue areas in Figure 3B).

### Resting State Functional MRI

**FIGURE 4:**
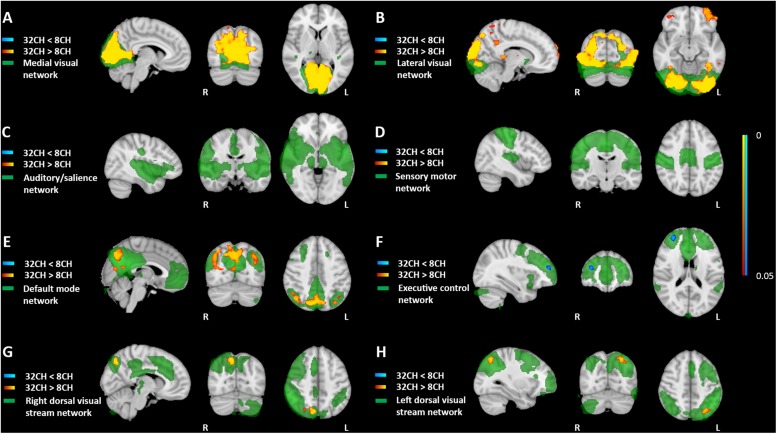
Voxel-based coil differences in network-based functional connectivity using dual regression paired sample *t*-test on resting state fMRI images. 8CH = 8 channel head coil, 32CH = 32 channel head coil. **(A–H)** Represents standard resting state networks of interest, illustrated and named in green. *p* values are color coded from 0.05 to <0.0001 FWE corrected.

Resting state functional connectivity was predominantly higher when using the 32CH coil compared with the 8CH coil (yellow areas in Figure 4), between the medial visual network and the lateral occipital cortex, calcarine cortex, and lingual gyrus (*p*^FWE^ < 0.05, effect size = 0.392, increase = 13.5%; Figure 4A), between the lateral visual network, the lateral occipital cortex and the occipital pole (*p*^FWE^ < 0.05, effect size = 0.637, increase = 27.5%; Figure 4B), between the default mode network and the lateral occipital cortex (*p*^FWE^ < 0.05, effect size = 0.505, increase = 9.4%; Figure 4E), and between the dorsal visual stream networks and regions of the lateral occipital cortex (right: *p*^FWE^ < 0.05, effect size = 0.368, increase = 13.6%; Figure 4G and left: *p*^FWE^ < 0.05, effect size = 0.391, increase = 12.04%; Figure 4H). Functional connectivity was lower with the 32CH coil in the executive control network and a small area in the frontal pole (*p*^FWE^ < 0.05, effect size = 0.608, decrease = 23.1%; blue area in Figure 4F). No differences in functional connectivity between both coils were found neither in the auditory, or salience network (Figure 4C), nor the sensory-motor network (Figure 4D).

### Voxel-Specific Scaling Factors

Results are described in detail in the Supplementary Material. For T1-weighted imaging, after applying the scaling factor, VBM analyses showed almost complete removal of the head coil differences throughout the brain for both GM and WM (Supplementary Figure S3). Validation of the DTI scaling factors showed a successful harmonization of the FA images, removing all significant coil differences. For MD, head coil differences were reduced, except for some small areas at the forceps major and right thalamus (Supplementary Figure S4). For resting state analyses, validation of the scaling factors showed extensive reduction of coil differences in the medial and lateral visual networks, and complete removal of all significant differences in the default mode network, executive control network (salience network), and the left and right dorsal visual stream networks (Supplementary Figure S5).

## Discussion

In this study, we demonstrated that quantitative results of standard processing pipelines for T1-weighted MRI, diffusion tensor imaging and resting state fMRI will be severely affected by the use of different head coils. Paired-wise group analyses between coils revealed different patterns of gray and white matter volume, white matter connectivity and functional connectivity.

Voxel-based morphometric analysis of T1-weighted imaging data, revealed smaller apparent gray matter volume for the 32CH coil in the outer frontal, temporal and parietal cortex, the inner cerebellum, the precuneus, and posterior cingulate cortex compared to the 8CH coil. Gray matter volume was larger using the 32CH coil in the occipital lobe, the central layer of the frontal, temporal, parietal cortex, the peripheral layer of the cerebellum, and subcortical areas. A less extensive but similar pattern of gray matter volume differences was previously found using two identical scanners with, respectively, an 8-channel and a 12-channel head coil (Focke et al., 2011). Compared to gray matter VBM, we found an opposite pattern of head coil differences for the white matter VBM, meaning that in areas where gray matter volume was larger, white matter volume was smaller and vice versa. The visual overlap of gray and white matter differences may be a result of the spatial smoothing (7 mm). When the contrast between gray and white matter is unclear, a higher level of smoothing is necessary to account for the uncertainties in partial volume estimation. Our results indicate that differences in gray/white matter contrast of the images leads to differences in tissue classification during segmentation (Zhang et al., 2001; Tohka, 2014). Three factors could have led to altered image contrast: (1) despite using inhomogeneity correction prior to segmentation, the SNR of the 8CH and 32CH coil has affected the probabilities of gray and white matter (Wiggins et al., 2006; Reiss-Zimmermann et al., 2013). For example, in our case, higher SNR in posterior cortical areas for the 32CH coil increased the probability of a voxel being white matter, and the same principle may explain the results in the frontal areas of the 8CH coil, (2) both coils have differences in signal distribution due to the coil geometry (Blamire, 2008) and the CLEAR algorithm does not fully correct for these (Yun et al., 2007), or (3) there has been a difference in the effective b1 distribution (Marques et al., 2010). As we demonstrate here, differences in image contrast in structural images could pose serious problems for studies combining MRI hardware elements and need to be equalized before tissue segmentation and partial volume estimation to prevent methodological errors.

We demonstrated that for DTI, measured FA was higher for nearly the entire white matter when comparing the 32CH coil with the 8CH coil, except for parts of the left temporal and parietal lobe. For MD, the results were hemispherical dependent, i.e., the 32CH coil showed higher MD compared to the 8CH coil in the white matter of the left hemisphere but lower MDs in the right hemisphere. The asymmetry in especially MD metrics was unexpected and inexplicable. Previous research demonstrated that MD may have more variance and be less reproducible than FA, even within sites (Fox et al., 2012; Jovicich et al., 2014; Helmer et al., 2016). Interestingly, the unexpected pattern in diffusion metrics was different from the SNR profiles of both coils and volumetric results. Therefore, we emphasize that SNR profiles and pattern of changes that occur in T1-weighted data cannot be translated to DTI data. Our results underline previous studies that already demonstrated the possible pitfalls of pooling diffusion data (Pagani et al., 2010; Vollmar et al., 2010; Takao et al., 2012; Venkatraman et al., 2015). Harmonization methods have been investigated in a number of studies, in attempt to overcome the problems with pooled DTI data, but sufficient harmonization has proven to be difficult (Mirzaalian et al., 2015; Venkatraman et al., 2015; Pohl et al., 2016; Fortin et al., 2017). Note that we slightly increased our FOV to allow coverage of the cerebellum for the 32CH coil data, which also increased TR, and could have influenced our results. This is, however, expected to be a very minor effect, since both TRs are significantly longer than five times the T1 of the white matter.

Resting state fMRI analyses showed increased functional connectivity between multiple posterior located networks and brains areas using the 32CH coil, corresponding to the SNR profile. Decreased functional connectivity using the 32CH coil was found between a small frontal area and the executive control network. Previous studies demonstrated around 10 percent variability between scanner hardware (Costafreda et al., 2007; Friedman et al., 2008; Kaza et al., 2011), but others could not detect significant differences in resting state networks between the 8CH and 32CH Philips head coils (Paolini et al., 2015). As our current sample size (*n* = 77) is larger than previous studies (*n* = 26), we assume that the increased power in our study allowed for detection of coil differences in resting state functional connectivity. Concordant with the results from our study, past research demonstrated that the 32CH coil has particularly increased SNR in posterior cortical areas compared to coils with less channels, caused by a difference in coil geometry (Wiggins et al., 2006; Reiss-Zimmermann et al., 2013). Resting state functional MRI studies may benefit from increased SNR in the 32CH coil, especially when posterior cortical areas are of interest.

Since changing head coils during longitudinal clinical research might introduce bias, we aimed to create voxel-based scaling images (Supplementary Material) for our own longitudinal clinical study (Dopper et al., 2014; Papma et al., 2017). The unique within-subject design in a large cohort (*n* = 77) with stable acquisition parameters allowed for the use of voxel-specific information for VBM, DTI, and resting state fMRI scaling. We validated the use of the scaling factors on independent data and demonstrated that coil differences can be substantially reduced when using voxel-based scaling in all modalities (see Supplementary Material). Our voxel-specific scaling factors may be an interesting harmonization method for within-subject variation. In previous literature, many harmonization methods have aimed to equalize neuroimaging data, all with their own advantages and shortcomings (Fennema-Notestine et al., 2007; Keihaninejad et al., 2010; Takao et al., 2011; Chen et al., 2014; Griffanti et al., 2014; Salimi-Khorshidi et al., 2014; Feis et al., 2015; Mirzaalian et al., 2015; Venkatraman et al., 2015; Pardoe et al., 2016; Pohl et al., 2016; Fortin et al., 2017; Li et al., 2018). For example, additional complexity in statistical models may cause decreased sensitivity for the actual outcome of interest (Li et al., 2018). Neuroimaging studies combining multiple MRI head coils and other hardware elements should be aware of confounding factors and be committed to use robust, sensitive and validated methods to deal with these factors (Jack et al., 2008; Smith and Nichols, 2018; Boeve and Rosen, 2019). We aim to deepen our research into neuroimaging harmonization methods and our scaling factors in the near future.

Major strengths of this study are the large sample size and the within-subject study design of two protocols within one scanning session using similar acquisition parameters. Despite our best efforts, some confounding factors are essentially inevitable, such as scanner drift, re-positioning of the participants heads inside the different coils and use of head cushions (Littmann et al., 2006). Other factors that could have influenced the results in our study may be habituation of the subject and scanner warm-up, especially for the resting state fMRI, and the anisotropic voxel size of the T1 weighted sequence. We are aware that extrapolating the results from our study to other head coils or other vendors may be difficult. Instead, we emphasize that our study may be appreciated as increasing awareness for the variability and possible bias in quantitative MRI metrics in data originating from different hardware elements. This is especially important for clinical research, where data acquired with different hardware elements is increasingly pooled.

In conclusion, this study provides evidence that the results of standard analysis models are severely compromised when data from different head coils is combined, or when head coils are changed during longitudinal clinical studies, even though acquisition protocols are completely harmonized. Studies combining neuroimaging MRI data with multiple head coils or other MRI hardware elements should be aware that measurements of gray and white matter volume, white matter connectivity and functional connectivity will differ between head coils and should handle these confounding factors with caution.

## Data Availability

The datasets for this manuscript are not publicly available yet as they are part of the ongoing longitudinal FTD-RisC study and the international GENFI cohort. Requests to access the raw MRI datasets should be directed to JS (j.c.vanswieten@erasmusmc.nl). Voxel-specific scaling factors from this study are available upon reasonable request to AH (a.hafkemeijer@lumc.nl).

## Ethics Statement

The study has been carried out in accordance with the Declaration of Helsinki and has been approved by the Medical and Ethical Review Committees of the Erasmus MC University Medical Center, Rotterdam, Netherlands and the Leiden University Medical Center, Leiden, Netherlands. Written informed consent has been obtained from all participants.

## Author Contributions

JLP designed the study, collected and analyzed the data, and was involved in data interpretation and writing of the manuscript. YT, MO, JG, and JMPa contributed to data analyses, interpretation and writing of the manuscript. EE, JMPo, LJ, LM, ED, and JS were involved in data collection and writing of the manuscript. MB and SR contributed to data interpretation and writing of the manuscript. AH was involved in study design, analyses and interpretation of the data, and writing of the manuscript.

## Conflict of Interest Statement

MO received funding from Philips, Best, the Netherlands. The funder was not involved in the study design, collection, analysis, interpretation of data, writing of the article or the decision to submit it for publication. The funder did permit publication of a figure depicting their hardware. The remaining authors declare that the research was conducted in the absence of any commercial or financial relationships that could be construed as a potential conflict of interest.

## References

[B1] Beckmann C. F. DeLuca M. Devlin J. T. Smith S. M. (2005). Investigations into resting-state connectivity using independent component analysis. *Philos. Trans R. Soc. Lond. B Biol. Sci.* 360 1001–1013. 10.1098/rstb.2005.1634 16087444 PMC1854918

[B2] Blamire A. M. (2008). The technology of MRI–the next 10 years? *Br. J. Radiol.* 81 601–617. 10.1259/bjr/96872829 18628329

[B3] Boeve B. F. Rosen H. J. (2019). Multimodal imaging in familial FTLD: phenoconversion and planning for the future. *Brain* 142 8–11. 10.1093/brain/awy314 30596906 PMC6308307

[B4] Cannon T. D. Sun F. McEwen S. J. Papademetris X. He G. van Erp T. G. (2014). Reliability of neuroanatomical measurements in a multisite longitudinal study of youth at risk for psychosis. *Hum. Brain Mapp.* 35 2424–2434. 10.1002/hbm.22338 23982962 PMC3843968

[B5] Chen J. Liu J. Calhoun V. D. Arias-Vasquez A. Zwiers M. P. Gupta C. N. (2014). Exploration of scanning effects in multi-site structural MRI studies. *J. Neurosci. Methods* 230 37–50. 10.1016/j.jneumeth.2014.04.023 24785589 PMC4114231

[B6] Costafreda S. G. Brammer M. J. Vencio R. Z. Mourao M. L. Portela L. A. de Castro C. C. (2007). Multisite fMRI reproducibility of a motor task using identical MR systems. *J. Magn. Reson. Imaging* 26 1122–1126. 10.1002/jmri.21118 17896376

[B7] Cummings J. L. Mega M. Gray K. Rosenberg-Thompson S. Carusi D. A. Gornbein J. (1994). The neuropsychiatric inventory: comprehensive assessment of psychopathology in dementia. *Neurology* 44 2308–2314. 7991117 10.1212/wnl.44.12.2308

[B8] Dopper E. G. Rombouts S. A. Jiskoot L. C. den Heijer T. de Graaf J. R. de Koning I. (2014). Structural and functional brain connectivity in presymptomatic familial frontotemporal dementia. *Neurology* 83 e19–e26. 10.1212/WNL.0000000000000583 25002573

[B9] Dubois B. Slachevsky A. Litvan I. Pillon B. (2000). The FAB: a frontal assessment battery at bedside. *Neurology* 55 1621–1626. 10.1212/wnl.55.11.1621 11113214

[B10] Feis R. A. Smith S. M. Filippini N. Douaud G. Dopper E. G. Heise V. (2015). ICA-based artifact removal diminishes scan site differences in multi-center resting-state fMRI. *Front. Neurosci.* 9:395. 10.3389/fnins.2015.00395 26578859 PMC4621866

[B11] Fennema-Notestine C. Gamst A. C. Quinn B. T. Pacheco J. Jernigan T. L. Thal L. (2007). Feasibility of multi-site clinical structural neuroimaging studies of aging using legacy data. *Neuroinformatics* 5 235–245. 10.1007/s12021-007-9003-9 17999200

[B12] Focke N. K. Helms G. Kaspar S. Diederich C. Toth V. Dechent P. (2011). Multi-site voxel-based morphometry–not quite there yet. *Neuroimage* 56 1164–1170. 10.1016/j.neuroimage.2011.02.029 21324367

[B13] Folstein M. F. Folstein S. E. McHugh P. R. (1975). Mini-mental state”. A practical method for grading the cognitive state of patients for the clinician. *J. Psychiatr. Res.* 12 189–198. 1202204 10.1016/0022-3956(75)90026-6

[B14] Fortin J. P. Parker D. Tunc B. Watanabe T. Elliott M. A. Ruparel K. (2017). Harmonization of multi-site diffusion tensor imaging data. *Neuroimage* 161 149–170. 10.1016/j.neuroimage.2017.08.047 28826946 PMC5736019

[B15] Fox R. J. Sakaie K. Lee J. C. Debbins J. P. Liu Y. Arnold D. L. (2012). A validation study of multicenter diffusion tensor imaging: reliability of fractional anisotropy and diffusivity values. *AJNR Am. J. Neuroradiol.* 33 695–700. 10.3174/ajnr.A2844 22173748 PMC8050446

[B16] Friedman L. Stern H. Brown G. G. Mathalon D. H. Turner J. Glover G. H. (2008). Test-retest and between-site reliability in a multicenter fMRI study. *Hum. Brain Mapp.* 29 958–972. 10.1002/hbm.20440 17636563 PMC3670112

[B17] Griffanti L. Salimi-Khorshidi G. Beckmann C. F. Auerbach E. J. Douaud G. Sexton C. E. (2014). ICA-based artefact removal and accelerated fMRI acquisition for improved resting state network imaging. *Neuroimage* 95 232–247. 10.1016/j.neuroimage.2014.03.034 24657355 PMC4154346

[B18] Haacke E. M. Brown R. W. Thompson M. R. Venkatesan R. (1999). *Magnetic Resonance Imaging: Physical Principles and Sequence Design.* Hoboken, NJ: Wiley.

[B19] Hafkemeijer A. Moller C. Dopper E. G. Jiskoot L. C. van den Berg-Huysmans A. A. van Swieten J. C. (2017). A Longitudinal study on resting state functional connectivity in behavioral variant frontotemporal dementia and Alzheimer’s Disease. *J. Alzheimers Dis.* 55 521–537. 10.3233/jad-150695 27662284

[B20] Helmer K. G. Chou M. C. Preciado R. I. Gimi B. Rollins N. K. Song A. (2016). Multi-site study of diffusion metric variability: characterizing the effects of site, vendor, field strength, and echo time using the histogram distance. *Proc. SPIE Int. Soc. Opt. Eng.* 9788:97881G. 10.1117/12.2217449 27350723 PMC4919981

[B21] Jack C. R. Jr. Bernstein M. A. Fox N. C. Thompson P. Alexander G. Harvey D. (2008). The Alzheimer’s Disease neuroimaging initiative (ADNI): MRI methods. *J. Magn. Reson. Imaging* 27 685–691. 10.1002/jmri.21049 18302232 PMC2544629

[B22] Jenkinson M. Beckmann C. F. Behrens T. E. Woolrich M. W. Smith S. M. (2012). Fsl. *Neuroimage* 62 782–790. 10.1016/j.neuroimage.2011.09.015 21979382

[B23] Jovicich J. Marizzoni M. Bosch B. Bartres-Faz D. Arnold J. Benninghoff J. (2014). Multisite longitudinal reliability of tract-based spatial statistics in diffusion tensor imaging of healthy elderly subjects. *Neuroimage* 101 390–403. 10.1016/j.neuroimage.2014.06.075 25026156

[B24] Kaza E. Klose U. Lotze M. (2011). Comparison of a 32-channel with a 12-channel head coil: are there relevant improvements for functional imaging? *J. Magn. Reson. Imaging* 34 173–183. 10.1002/jmri.22614 21618334

[B25] Keihaninejad S. Heckemann R. A. Fagiolo G. Symms M. R. Hajnal J. V. Hammers A. (2010). A robust method to estimate the intracranial volume across MRI field strengths (1.5T and 3T). *Neuroimage* 50 1427–1437. 10.1016/j.neuroimage.2010.01.064 20114082 PMC2883144

[B26] Li H. Smith S. M. Gruber S. Lukas S. E. Silveri M. M. Hill K. P. (2018). Combining multi-site/multi-study MRI DATA: linked-ICA denoising for removing scanner and site variability from multimodal MRI data. *bioRxiv*

[B27] Littmann A. Guehring J. Buechel C. Stiehl H. S. (2006). Acquisition-related morphological variability in structural MRI. *Acad. Radiol.* 13 1055–1061. 10.1016/j.acra.2006.05.001 16935717

[B28] Marques J. P. Kober T. Krueger G. van der Zwaag W. Van de Moortele P. F. Gruetter R. (2010). MP2RAGE, a self bias-field corrected sequence for improved segmentation and T1-mapping at high field. *Neuroimage* 49 1271–1281. 10.1016/j.neuroimage.2009.10.002 19819338

[B29] Mioshi E. Hsieh S. Savage S. Hornberger M. Hodges J. R. (2010). Clinical staging and disease progression in frontotemporal dementia. *Neurology* 74 1591–1597. 10.1212/WNL.0b013e3181e04070 20479357

[B30] Mirzaalian H. de Pierrefeu A. Savadjiev P. Pasternak O. Bouix S. Kubicki M. (2015). Harmonizing diffusion mri data across multiple sites and scanners. *Med. Image Comput. Comput. Assist. Interv.* 9349 12–19. 10.1007/978-3-319-24553-9_2 27754499 PMC5045042

[B31] Nichols T. E. Holmes A. P. (2002). Nonparametric permutation tests for functional neuroimaging: a primer with examples. *Hum. Brain Mapp.* 15 1–25. 10.1002/hbm.1058 11747097 PMC6871862

[B32] Pagani E. Hirsch J. G. Pouwels P. J. Horsfield M. A. Perego E. Gass A. (2010). Intercenter differences in diffusion tensor MRI acquisition. *J. Magn. Reson. Imaging* 31 1458–1468. 10.1002/jmri.22186 20512899

[B33] Paolini M. Keeser D. Ingrisch M. Werner N. Kindermann N. Reiser M. (2015). Resting-state networks in healthy adult subjects: a comparison between a 32-element and an 8-element phased array head coil at 3.0 Tesla. *Acta Radiol.* 56 605–613. 10.1177/0284185114567703 25585849

[B34] Papma J. M. Jiskoot L. C. Panman J. L. Dopper E. G. den Heijer T. Donker Kaat L. (2017). Cognition and gray and white matter characteristics of presymptomatic C9orf72 repeat expansion. *Neurology* 89 1256–1264. 10.1212/WNL.0000000000004393 28855404

[B35] Pardoe H. R. Cutter G. R. Alter R. Hiess R. K. Semmelroch M. Parker D. (2016). Pooling morphometric estimates: a statistical equivalence approach. *J. Neuroimaging* 26 109–115. 10.1111/jon.12265 26094850

[B36] Pohl K. M. Sullivan E. V. Rohlfing T. Chu W. Kwon D. Nichols B. N. (2016). Harmonizing DTI measurements across scanners to examine the development of white matter microstructure in 803 adolescents of the NCANDA study. *Neuroimage* 130 194–213. 10.1016/j.neuroimage.2016.01.061 26872408 PMC4808415

[B37] Pruim R. H. R. Mennes M. Buitelaar J. K. Beckmann C. F. (2015). Evaluation of ICA-AROMA and alternative strategies for motion artifact removal in resting state fMRI. *Neuroimage* 112 278–287. 10.1016/j.neuroimage.2015.02.063 25770990

[B38] Reiss-Zimmermann M. Gutberlet M. Kostler H. Fritzsch D. Hoffmann K. T. (2013). Improvement of SNR and acquisition acceleration using a 32-channel head coil compared to a 12-channel head coil at 3T. *Acta Radiol.* 54 702–708. 10.1177/0284185113479051 23474767

[B39] Salimi-Khorshidi G. Douaud G. Beckmann C. F. Glasser M. F. Griffanti L. Smith S. M. (2014). Automatic denoising of functional MRI data: combining independent component analysis and hierarchical fusion of classifiers. *Neuroimage* 90 449–468. 10.1016/j.neuroimage.2013.11.046 24389422 PMC4019210

[B40] Smith S. M. Jenkinson M. Johansen-Berg H. Rueckert D. Nichols T. E. Mackay C. E. (2006). Tract-based spatial statistics: voxelwise analysis of multi-subject diffusion data. *Neuroimage* 31 1487–1505. 10.1016/j.neuroimage.2006.02.024 16624579

[B41] Smith S. M. Nichols T. E. (2018). Statistical challenges in “big data”. *Hum. Neuroimaging. Neuron* 97 263–268. 10.1016/j.neuron.2017.12.018 29346749

[B42] Takao H. Hayashi N. Kabasawa H. Ohtomo K. (2012). Effect of scanner in longitudinal diffusion tensor imaging studies. *Hum. Brain Mapp.* 33 466–477. 10.1002/hbm.21225 21391276 PMC6869949

[B43] Takao H. Hayashi N. Ohtomo K. (2011). Effect of scanner in longitudinal studies of brain volume changes. *J. Magn. Reson. Imaging* 34 438–444. 10.1002/jmri.22636 21692137

[B44] Tohka J. (2014). Partial volume effect modeling for segmentation and tissue classification of brain magnetic resonance images: a review. *World J. Radiol.* 6 855–864. 25431640 10.4329/wjr.v6.i11.855 PMC4241492

[B45] Venkatraman V. K. Gonzalez C. E. Landman B. Goh J. Reiter D. A. An Y. (2015). Region of interest correction factors improve reliability of diffusion imaging measures within and across scanners and field strengths. *Neuroimage* 119 406–416. 10.1016/j.neuroimage.2015.06.078 26146196 PMC5519407

[B46] Verhage F. (1964). *Intelligence and Age: Study with Dutch People Aged 12–77 (in Dutch).* Assen: van Gorcum.

[B47] Vollmar C. O’Muircheartaigh J. Barker G. J. Symms M. R. Thompson P. Kumari V. (2010). Identical, but not the same: intra-site and inter-site reproducibility of fractional anisotropy measures on two 3.0T scanners. *Neuroimage* 51 1384–1394. 10.1016/j.neuroimage.2010.03.046 20338248 PMC3163823

[B48] Wiggins G. C. Triantafyllou C. Potthast A. Reykowski A. Nittka M. Wald L. L. (2006). 32-channel 3 Tesla receive-only phased-array head coil with soccer-ball element geometry. *Magn. Reson. Med.* 56 216–223. 10.1002/mrm.20925 16767762

[B49] Yun S. Kyriakos W. E. Chung J. Y. Han Y. Yoo S. S. Park H. (2007). Projection-based estimation and nonuniformity correction of sensitivity profiles in phased-array surface coils. *J. Magn. Reson. Imaging* 25 588–597. 10.1002/jmri.20826 17326086

[B50] Zhang Y. Brady M. Smith S. (2001). Segmentation of brain MR images through a hidden Markov random field model and the expectation-maximization algorithm. *IEEE Trans. Med. Imaging* 20 45–57. 10.1109/42.906424 11293691

