# Supplementary figures and images for: Bias Introduced by Multiple Head Coils in MRI Research: An 8 Channel and 32 Channel Coil Comparison
Source: Front Neurosci. 2019 Jul 15;13:729. doi: 10.3389/fnins.2019.00729 (PMC6648353; doi:10.3389/fnins.2019.00729)

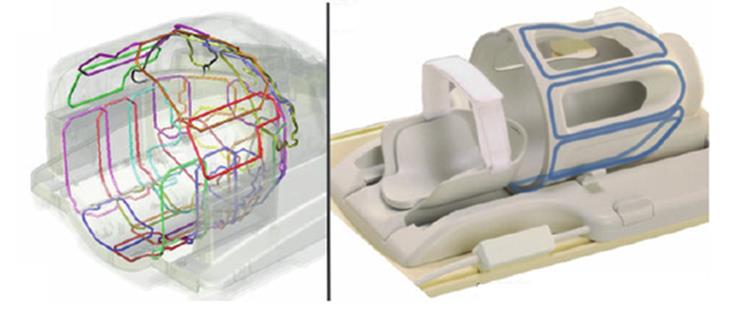

Supplement: Supplementary file 1 [file Image_1.JPEG]

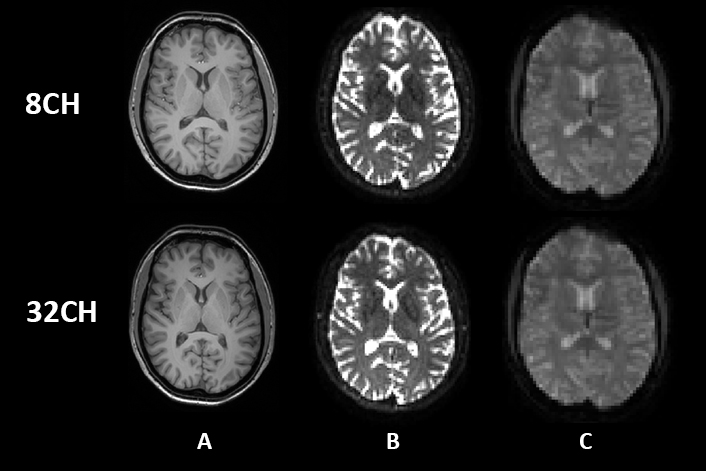

Supplement: Supplementary file 2 [file Image_2.JPEG]

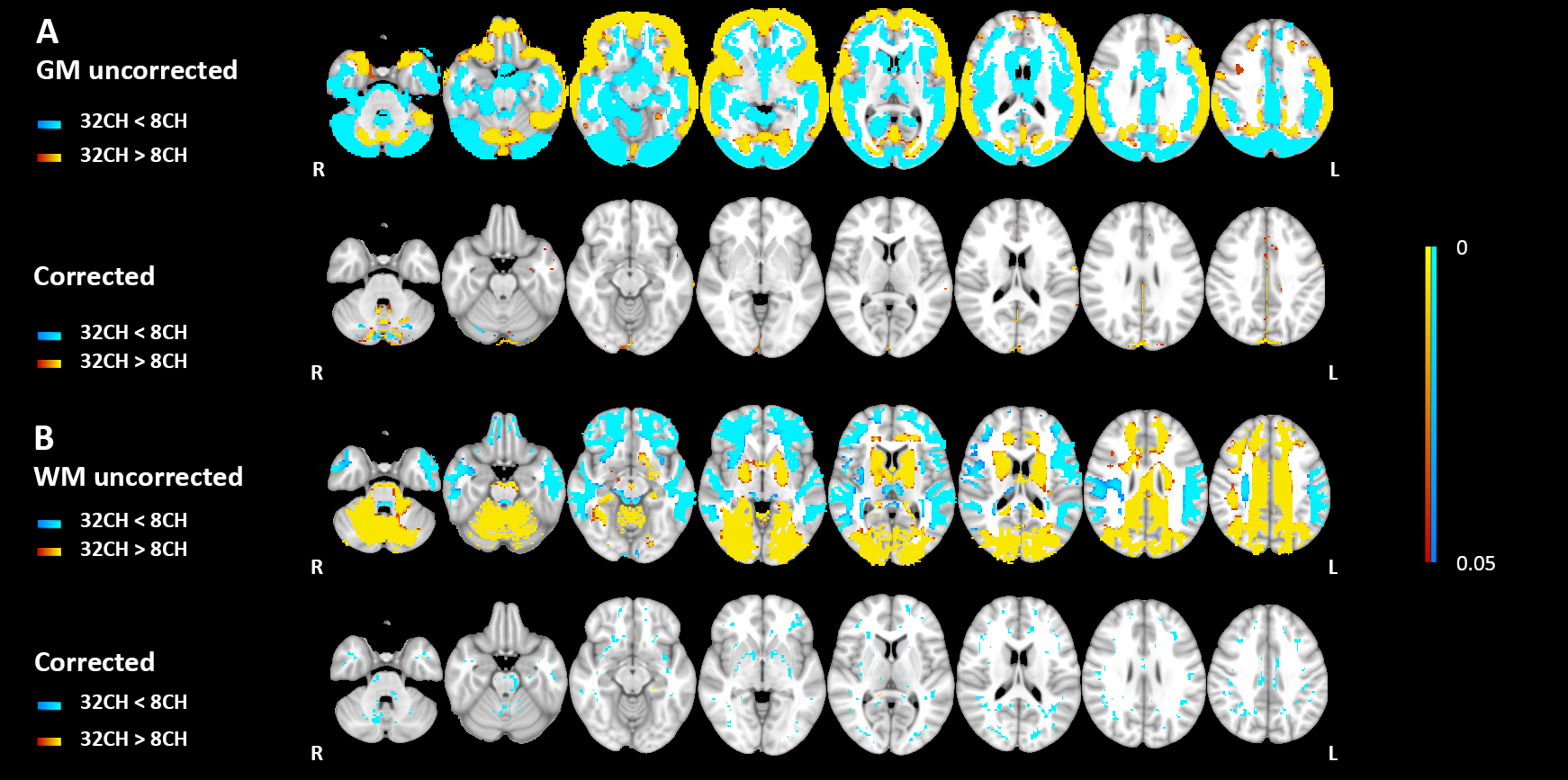

Supplement: Supplementary file 3 [file Image_3.JPEG]

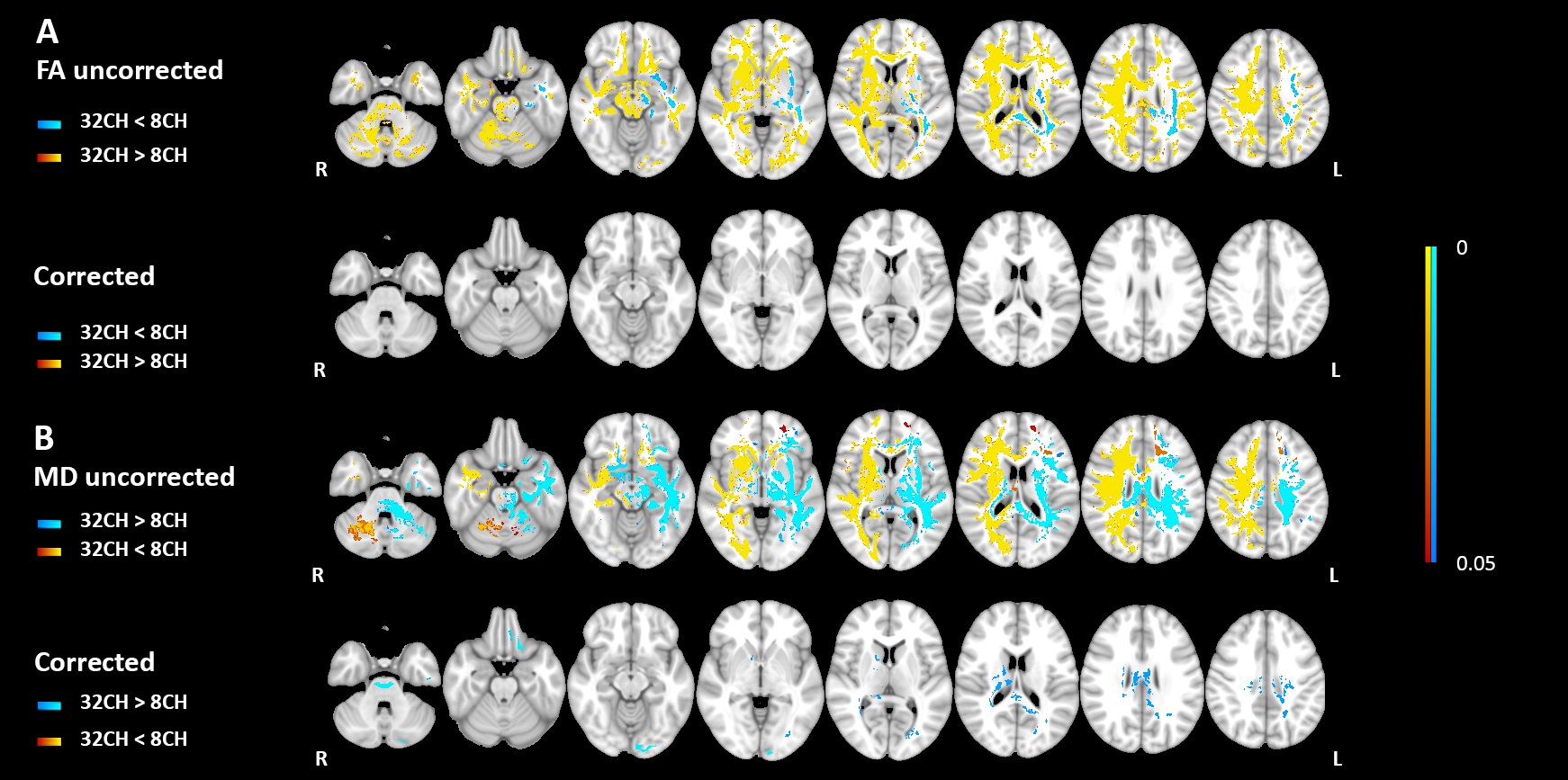

Supplement: Supplementary file 4 [file Image_4.JPEG]

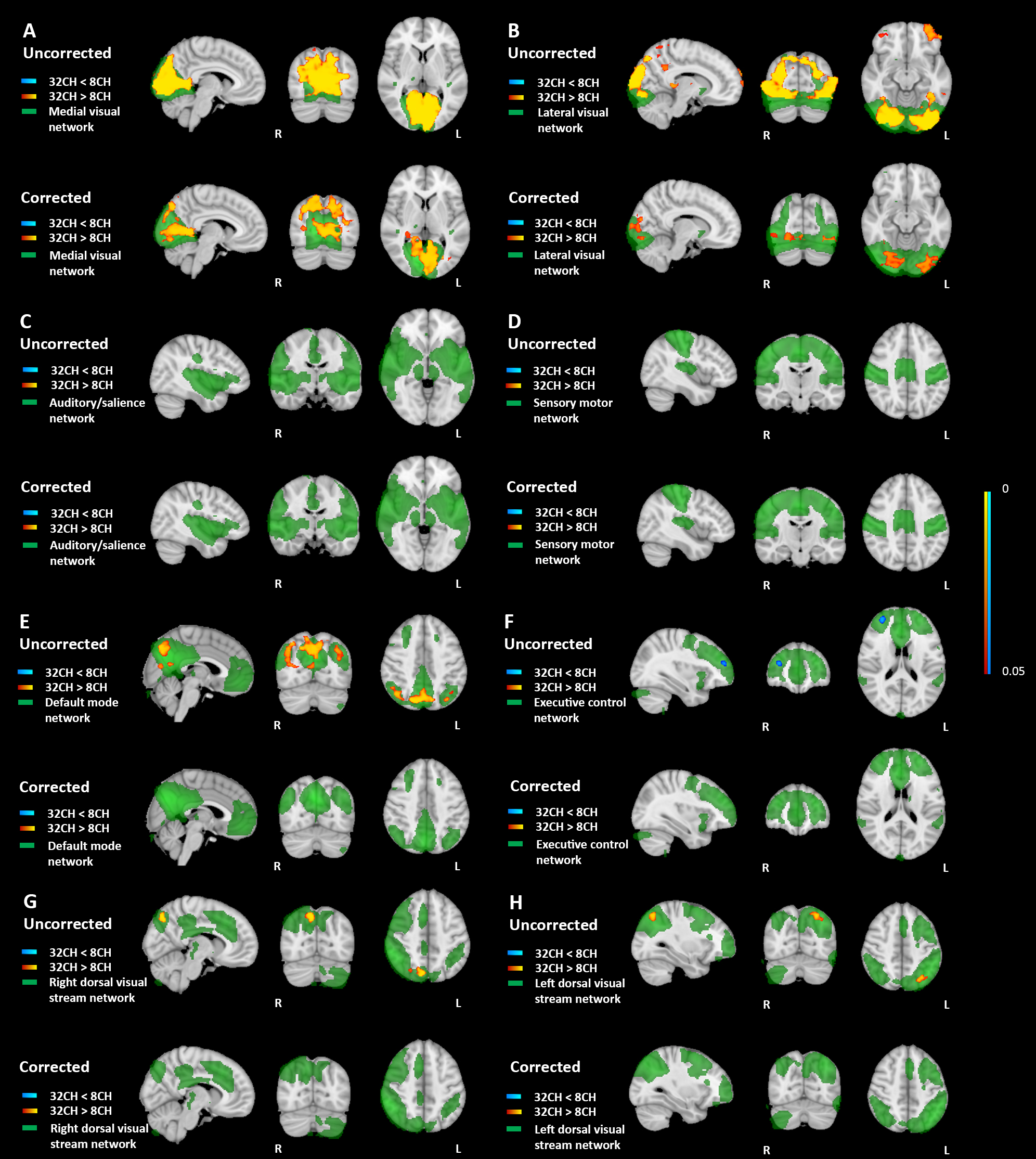

Supplement: Supplementary file 5 [file Image_5.JPEG]
