# Supplementary material for: Bias Introduced by Multiple Head Coils in MRI Research: An 8 Channel and 32 Channel Coil Comparison
Source: Front Neurosci. 2019 Jul 15;13:729. doi: 10.3389/fnins.2019.00729 (PMC6648353; doi:10.3389/fnins.2019.00729)
Supplement: Supplementary file 6 [file Data_Sheet_1.docx]

**SUPPLEMENTARY MATERIAL**

**Coil geometry**


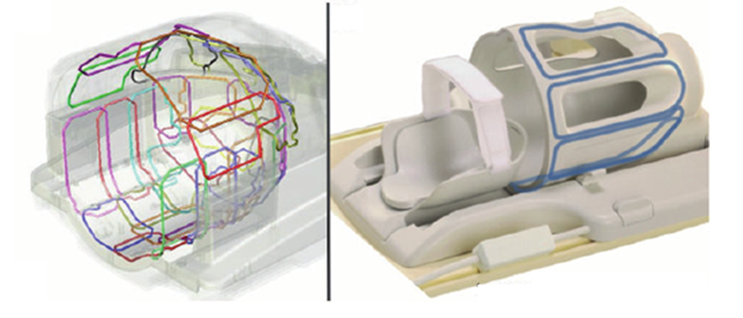


**Supplementary Figure 1.** Coil geometry for the 32 channel head coil (left) and the 8 channel head coil (right). Inside diameter at the opening is 26 cm for the 32 channel and 25.5 cm for the 8 channel head coil. Reprinted with permission from Philips, Best, The Netherlands.

**Raw image types examples**

**
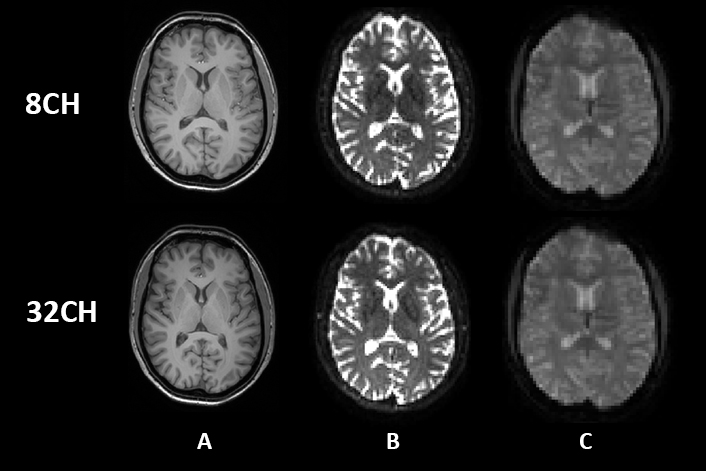
**

**Supplementary Figure 2.** Raw image types from 8 channel head coil and 32 channel head coil for one representative healthy participant. A: 3DT1-weighted, B: b0 image from DTI, C: BOLD EPI image from resting state fMRI.

**Scaling factor**

1. **Methods**

To correct for the use of different head coils (8CH versus 32CH), we defined voxel-based scaling factors for T1-weighted grey matter and white matter segmentations, for FA and MD images derived from DTI and for time-course data from the resting state fMRI images. First, we separated the sample into two datasets, matched for age and sex, into a ‘template’ dataset (n=39) and a ‘validation’ dataset (n=38). The template dataset was used to create the scaling factor, and in order to validate it, we applied the scaling factor to the validation dataset.

For the template dataset, each subject’s images from the 8CH coil and their corresponding 32CH coil images were merged and averaged into a separate mean 8CH coil image and a mean 32CH coil image. Subsequently, the mean 32CH coil was divided by the mean 8CH coil image, resulting in a voxel-based scaling factor. The scaling factor was reduced from noise using median filtering with a kernel of 5 mm.

For validation purposes, we multiplied images from the 8CH coil in the validation dataset with the scaling factor, equalizing the 8CH coil images to the signal intensity of the 32CH coil images in the validation dataset. In order to confirm successful usage of the scaling factor, voxel-based statistical testing with the corrected 8CH coil images and the original 32CH coil images of the validation dataset was repeated using the previous described analysis steps for the T1, DTI and resting state fMRI images.

Finally, we created the voxel-based scaling factors using the full dataset (n=77), to correct for head coil differences in our current longitudinal FTD-RisC study. These scaling factors are available for other researchers upon request.

1. **Results**

**2.1. T1-weighted**

Before applying the scaling factor, VBM analysis in the validation dataset (n=38) showed the same differences in grey and white matter between head coils as when using the full dataset (n=77) (Supplementary Figure 3, uncorrected). After applying the scaling factor, the VBM analyses showed extensive reduction of the head coil differences throughout the brain for both GM and WM (Supplementary Figure 3, corrected). For GM, head coil differences remained present in only a small superior part of the cerebellum (Supplementary Figure 3, GM corrected). For WM, the head coil differences were reduced after applying the scaling factor, except some small areas on the grey-white matter border in the brain (Supplementary Figure 3, WM corrected).

**2.2. Diffusion tensor imaging**

Coil differences in FA and MD, before applying the scaling factors, were similar in the validation dataset (n=38) compared with the coil differences found using the full dataset (n=77) (Supplementary Figure 4, uncorrected). Validation of the DTI scaling factors showed a successful harmonization of the FA images, removing all significant coil differences (Supplementary Figure 4, FA corrected). For MD, head coil differences were reduced, except for some small areas at the forceps major and right thalamus (Supplementary Figure 4, MD corrected).

**2.3. Resting state functional MRI**

Before applying the scaling factors, coil differences in functional connectivity were similar in the validation dataset (n=38) compared with the differences found using the full dataset (n=77) (Supplementary Figure 5, A-H, uncorrected). After applying the scaling factor, the resting state analyses showed extensive reduction in functional connectivity coil differences in the medial and lateral visual system networks (Supplementary Figure 5, networks A and B, corrected). The validation of the resting state scaling factors showed successful harmonization and removed all significant coil differences for the default mode network (Supplementary Figure 5, E, corrected), executive control network (also referred to as the salience network) (Supplementary Figure 5, F, corrected), and the left and right dorsal visual stream networks (Supplementary Figure 5, networks G and H, corrected).

**Supplementary Figure 3.** A) Voxel-based coil differences in grey matter volume (GM) before (uncorrected) and after applying the scaling factor to the validation dataset (corrected). B) Voxel-based coil differences in white matter volume (WM uncorrected) and remaining coil differences after applying the scaling factor to the validation dataset (corrected). P values are color coded from *0.05 to < 0.0001* FWE corrected.

**Supplementary Figure 4.** A) Voxel-based coil differences in fractional anisotropy (FA) before (uncorrected) and after applying the scaling factor to the validation dataset (corrected). B) Voxel-based coil differences in mean diffusivity (MD) before (uncorrected) and after applying the scaling factor to the validation dataset (corrected). P values are color coded from *0.05 to < 0.0001* FWE corrected.

**Supplementary Figure 5.** Voxel-based coil differences in network-based functional connectivity. Resting state networks of interest are indicated in green (A-H). Coil differences in functional connectivity before (uncorrected) and after applying the scaling factor to the validation dataset (corrected). P values are color coded from *0.05 to < 0.0001* FWE corrected.
